# Assessing sleep-wake survival dynamics in relation to sleep quality in a placebo-controlled pharmacological intervention study with people with insomnia and healthy controls

**DOI:** 10.1007/s00213-020-05660-3

**Published:** 2020-09-17

**Authors:** Lieke W. A. Hermans, Marta Regis, Pedro Fonseca, Sebastiaan Overeem, Tim R. M. Leufkens, Annemiek Vermeeren, Merel M. van Gilst

**Affiliations:** 1 Department of Electrical Engineering, Eindhoven University of Technology, De Zaale, Eindhoven, The Netherlands; 2 Department of Mathematics and Computer Science, Eindhoven University of Technology, De Zaale, Eindhoven, The Netherlands; 3 Philips Research, High Tech Campus 34, Eindhoven, The Netherlands; 4 Sleep Medicine Center Kempenhaeghe, Sterkselseweg 65, Heeze, The Netherlands; 5 Department of Neuropsychology and Psychopharmacology, Faculty of Psychology and Neuroscience, Maastricht University, Universiteitssingel 40, Maastricht, The Netherlands

**Keywords:** Insomnia, Sleep state misperception, Sleep onset latency, Sleep fragmentation, Zopiclone

## Abstract

**Rationale:**

The mechanisms underlying impaired sleep quality in insomnia are not fully known, but an important role for sleep fragmentation has been proposed.

**Objectives:**

The aim of this study is to explore potential mechanisms of sleep fragmentation influencing alterations of perceived sleep quality.

**Methods:**

We analyzed polysomnography (PSG) recordings from a double-blind crossover study with zopiclone 7.5 mg and placebo, in elderly participants with insomnia complaints and age-matched healthy controls. We compared survival dynamics of sleep and wake across group and treatment. Subsequently, we used a previously proposed model to estimate the amount of sleep onset latency (SOL) misperception from PSG-defined sleep fragmentation. Self-reported and model-estimated amount of SOL misperception were compared across group and treatment, as well as model prediction errors.

**Results:**

In the zopiclone night, the average segment length of NREM sleep was increased (group F = 1.16, *p* = 0.32; treatment F = 8.89, ***p*** **< 0.01**; group x treatment F = 0.44, *p* = 0.65), while the segment length of wake was decreased (group F = 1.48, *p* = 0.23; treatment F = 11.49, ***p*** **< 0.01**; group x treatment F = 0.36, *p* = 0.70). The self-reported and model-estimated amount of SOL misperception were lower during the zopiclone night (self-reported group F = 6.08, ***p*** **< 0.01**, treatment F = 10.8, ***p*** **< 0.01**, group x treatment F = 2.49, *p* = 0.09; model-estimated F = 1.70, *p* = 0.19, treatment F = 16.1, ***p*** **< 0.001**, group x treatment F = 0.60, *p* = 0.55). The prediction error was not altered (group F = 1.62, *p* = 0.20; treatment F = 0.20, *p* = 0.65; group x treatment F = 1.01, *p* = 0.37).

**Conclusions:**

Impaired subjective sleep quality is associated with decreased NREM stability, together with increased stability of wake. Furthermore, we conclude that zopiclone-induced changes in SOL misperception can be largely attributed to predictable changes of sleep architecture.

## Introduction

Despite ongoing research, part of the etiology of insomnia is still unknown. When considering insomnia as a medical problem with insufficient sleep, it seems likely that the sleep complaints could be objectively quantified using gold standard polysomnography (PSG) recordings. However, often standard PSG-derived metrics such as total sleep time (TST), sleep onset latency (SOL), and wake after sleep onset (WASO) do not fully explain the seriousness of the complaints (Baglioni et al. 2013). Specifically, in part of the patients, a discrepancy can be found between the amount of sleep reported by the patient and the objectively measured quantity of the sleep (Harvey and Tang 2013). Additionally, the experienced sleep quality is often also not reflected by standard PSG metrics (Svetnik et al. 2019). Therefore, it is assumed that impaired sleep can possibly be reflected by other PSG-derived parameters. Identifying such parameters could be useful to increase our understanding of the mechanisms underlying insomnia. Furthermore, they could potentially be used for identifying clinically meaningful subtypes within the patient population.

One of the objectively measurable sleep characteristics reflecting sleep quality may be impaired sleep continuity as scored in the hypnogram, which we here refer to as sleep fragmentation (Bonnet and Arand 2003; ^,^Stepanski et al. 1984; Wei et al. 2017; Harvey and Tang 2013). Interruptions of sleep at the beginning of the night may influence the perception of the SOL, since the sensation of being asleep prior to awakening from non-rapid eye movement (NREM) sleep was shown to depend on the length of the preceding bout of uninterrupted sleep (Sewitch 1984; Bonnet and Moore 1982; Hauri and Olmstead 1983). In previous research, we quantified the relationship between sleep fragmentation at the beginning of the night and sleep onset (mis)perception and found that the perception of the sleep onset latency indeed seems particularly influenced by the length of uninterrupted sleep fragments (Hermans et al. 2020a). Additionally, the objective and subjective numbers of awakenings have been shown to be correlated with measures of subjective sleep quality (Rosipal et al. 2013; Kaplan et al. 2018), providing an additional indication that sleep fragmentation may negatively influence sleep. However, it should be noted that the predictive power of objective sleep parameters for subjective sleep quality is generally low (Rosipal et al. 2013; Goelema et al. 2017).

Assessing the influence of sleep fragmentation on the experience of sleep presents two problems. First, it is likely that perceived quantity and quality of the sleep are influenced by many other factors than sleep structure, possibly including sleep habits, psychological traits (Freedman and Sattler 1982), and time estimation ability (Hermans et al. 2020b; Tang and Harvey 2005). Therefore, large variability between individuals can be expected. This variability could possibly obscure the relationship between perceived sleep quality and sleep fragmentation. Although currently we do not have the possibility to quantify and correct for these factors, they probably differ largely between people and may have a smaller variability over consecutive nights within the same individual. We would therefore ideally study multiple nights measured from the same individual, with induced differences of sleep architecture. Hypnotics are known to alter sleep structure and can improve the subjective experience of the sleep (Borbely et al. 1985; Hemmeter et al. 2000; Kryger et al. 1991). Therefore, medication studies can be useful to explore mechanisms of sleep fragmentation potentially influencing sleep quality under controlled circumstances. Second, currently there is no single parameter that is best suited for describing sleep fragmentation.

Survival analysis is potentially a very useful alternative for describing the aspects of sleep fragmentation which may be important for perceiving a good night of sleep. Traditional parameters used to describe sleep fragmentation as scored in the hypnogram include WASO, number of awakenings, and sleep stage percentages, such as NREM1 and NREM3 (Bianchi et al. 2012). These parameters are not very specific. As an illustration, Norman et al. showed that a large number of awakenings can be found in two entirely different types of sleep architecture (Norman et al. 2006). For example, awakenings regularly distributed over the night would result in sleep fragments of equal lengths, while the same number of clustered awakenings could also result in one very long sleep fragment and multiple short sleep fragments. Such differences can be found using survival analysis. Considering that participants appeared to overlook short sleep fragments in previous research into sleep onset misperception (Hermans et al. 2020a), such differences in sleep architecture may be important to take into account. Additionally, low percentages of certain sleep stages can either reflect the presence of many interrupted sleep stage fragments or a reduced probability to enter that sleep stage (Bianchi et al. 2012). Survival analysis can be used to specifically assess the stability of a certain sleep stage or groups of sleep stages. Using survival analysis, one can analyze the expected time until a certain new event occurs. In the case of sleep, the event can be the end of a sleep or wake fragment. A hazard function can be calculated to evaluate the probability of ending a sleep or wake fragment at any given time point. Or similarly, a survival function can be calculated to evaluate the probability of a fragment to survive.

In earlier research, Roth et al. showed differences in sleep survival dynamics between healthy people and people with insomnia (Roth et al. 2016). This finding indicates that altered sleep survival dynamics may indeed be involved in impaired sleep quality in people with insomnia. Rapid eye movement (REM) and NREM sleep were not separately modelled in this study, although research indicates that NREM and REM sleep are two different processes with different survival curve dynamics (Klerman et al. 2013) and different functions (Vyazovskiy and Delogu 2014). Moreover, it might be useful to also study survival dynamics of wake, because earlier research suggests that patients with long WASO belong to a distinct subtype of insomnia (Miller et al. 2016). Therefore, the next step is to examine if subjectively impaired sleep co-occurs with changes of sleep and wake survival dynamics during the night while separately assessing survival dynamics of NREM sleep, REM sleep, and wake.

Co-occurring changes in sleep fragmentation and perceived sleep quality can be very informative, but do not prove that these two phenomena really influence each other. Our earlier described modelling approach for sleep onset misperception can be used to quantify this relationship. This so-called sleep length model was based on the assumption that sleep bouts with a too short length at sleep onset are perceived as wake (Hermans et al. 2020a). Using the model, perceived sleep onset can be estimated as the start of the first sleep fragment longer than L minutes. Sleep length parameter L was the estimated parameter of the model, i.e., the length a continuous sleep fragment should have in order to be perceived as sleep. This concept is similar to calculating latency until persistent sleep. Applying this model yields a decomposition of sleep onset misperception into a component that can be predicted based on sleep fragmentation at the beginning of the night and an unexplained component. We can use such model to test if any change in sleep onset (mis)perception as a consequence of taking sleep medication can be related to alterations in the explained component of sleep fragmentation.

The aim of this study was to explore potential mechanisms of sleep fragmentation that influence alterations of perceived sleep quality. We analyze the data from a previously described placebo-controlled study, using a single dose of zopiclone 7.5 mg as experimental intervention (Leufkens et al. 2014). In the initial article, the authors reported that the subjective sleep quality was improved during the zopiclone night and that participants reported longer TST and shorter SOL. In the current study, we assessed the influence of zopiclone on survival dynamics of NREM sleep, REM sleep, and wake over the whole night. This way, we can examine if the improvements of sleep quality described co-occur with alterations of sleep fragmentation. Furthermore, we aim to demonstrate the relation between sleep architecture and perceived sleep quality, using a modelling approach.

## Methods

### Design

Data were collected as part of a placebo-controlled crossover study, comparing the residual effects of zopiclone 7.5 mg and placebo on highway driving performance in people with complaints of insomnia and self-defined good sleepers (Leufkens et al. 2014). Study participants included 16 individuals with insomnia complaints who frequently used hypnotics, 16 individuals with insomnia complaints who did not or infrequently used hypnotics, and 16 age-matched self-defined good sleepers.

### Participants

Participants were recruited via newspaper advertisements and through a network of local general practitioners in the region of Maastricht, the Netherlands (Leufkens et al. 2014), and were subsequently asked to participate in the placebo-controlled crossover zopiclone study. Participants had to meet the following inclusion criteria: aged between 50 and 75 years and good health based on a pre-study physical examination, medical history, vital signs, electrocardiogram, blood biochemistry, hematology, serology, and urinalysis. The exclusion criteria were history of drug or alcohol abuse; the presence of a significant medical, neurological, and psychiatric disorder, or sleep disorder other than insomnia; chronic use of medication that affects driving performance, except hypnotics; drinking more than 6 cups of coffee per day; drinking more than 21 units of alcohol per week; smoking more than 10 cigarettes per day; and body mass index outside the range of 19 to 30 kg/m^2^.

Additionally, patients with insomnia had to meet the following inclusion criteria, based on DSM-IV^28^: (1) presence of subjective complaints of insomnia, defined as difficulties initiating sleep (sleep latency > 30 min) and/or maintaining sleep (awakenings > 30 min); (2) complaints lasting more than 1 month; (3) clinically significant distress or impairment attributable to the sleep disturbance; (4) insomnia not occurring exclusively during the course of a mental disorder; and (5) insomnia not due to another medical or sleep disorder or to the effect of medication or drug abuse. Patients with insomnia were assigned to the “frequent users” group when they used a benzodiazepine, zopiclone, or zolpidem as sleeping medication for at least four nights per week during at least 3 months preceding the study. Patients not using hypnotics or using hypnotics for less than 4 days per week were assigned to the “infrequent users” group.

Volunteers were screened by a telephone interview, questionnaires, and a physical examination to confirm that they were healthy. Sleep complaints were evaluated by a trained psychologist using Dutch versions of the Pittsburgh Sleep Quality Index (Buysse et al. 1989), the Sleep Wake Experience List (Van Diest et al. 1989), and the Groningen Sleep Quality Scale (Mulder-Hajonides Van Der Meulen 1981). In addition, subjects completed a sleep log for 14 days. Major psychopathology was screened using the Symptom Checklist 90 Revised (Derogatis 1983), the Beck Depression Inventory (Beck et al. 1961), the State-Trait Anxiety Inventory (Spielberger et al. 1983), and the Multidimensional Fatigue Inventory (Smets et al. 1995).

The study was conducted in accordance with the code of ethics on human experimentation established by the World Medical Association’s Declaration of Helsinki (1964) and amended in Edinburgh (2000). The protocol was approved by the medical ethics committee of Maastricht University and University Hospital of Maastricht. Participants were explained the aims, methods, and potential hazards of the study and they signed a written informed consent prior to any study-related assessments.

### Schedule

The study was conducted according to a 3 × 2 double-blind, placebo-controlled crossover design, with three groups (insomnia frequently using hypnotics, insomnia not or infrequently using hypnotics, and self-defined good sleepers) and two treatment conditions. Treatments were single oral doses of zopiclone 7.5 mg and placebo. Treatments were administered in identical looking capsules and ingested immediately before retiring to bed at 23:30 h. All participants went to bed at the same fixed bedtime. Prior to the measurement nights, participants spent two nights in the same sleep laboratory. Treatment orders (placebo–zopiclone or vice versa) were balanced within groups. Washout periods between treatments lasted at least 1 week. In order to minimize withdrawal symptoms during the placebo night, patients assigned to the frequent users group were instructed to discontinue their hypnotic intake starting from three nights before each treatment period. Frequent users who expected difficulties during the three hypnotic-free nights were provided escape medication, consisting of zolpidem at a maximum of one dose of 10 mg per night, to be used only in case of intolerable withdrawal effects. Zolpidem 10 mg was selected to limit variability in hypnotic drugs used and because it is known to be free from residual effects when taken at bedtime before 8 h of sleep (Vermeeren 2004).

### Assessments

A four-channel electroencephalogram (C3, C4, F4, O2), electrooculogram, and electromyogram were performed as part of the polysomnographic acquisition. The data was recorded with a Vitaport portable EEG recorder with a common average (A1–A2) and a sample frequency of 256 Hz. Visual sleep staging was performed according to R&K criteria (Rechtschaffen and Kales 1968) by experienced technicians from the sleep center of Stichting Epilepsie Instellingen Nederland (Zwolle, the Netherlands). Technicians were blinded for the group affiliations of the subjects. Each polysomnogram was scored by one—the same—technician.

Subjective sleep measures were assessed the morning after the PSG measurements by asking subjects to report their subjective TST, SOL, number of awakenings, and time of early awakening.

### Survival analysis

**Fig. 1:**
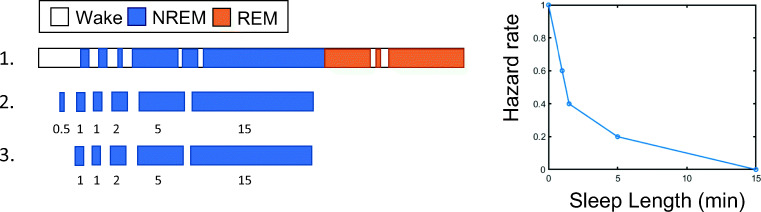
Illustration of survival curve analysis of NREM sleep. Step 1 depicts an example of sleep over time. For reasons of clarity, only one sleep cycle is depicted. During step 2, all fragments of NREM sleep are listed and sorted based on length. Fragments of NREM sleep are assumed to be terminated if they are followed by either wake or REM sleep. In step 3, NREM fragments shorter than 1 min are excluded from the analysis. On the right, the hazard rate resulting from these sleep fragments is plotted. The survival rate represents the percentage of NREM fragments longer than a certain length, e.g., after 1 min, three (60%) out of five of the sleep fragments are still left (i.e., “has survived”), and after 5 min, only one (20%) sleep fragment is left. A Weibull curve was fit to the hazard rate (not indicated in the figure). Survival curves of REM sleep and wake sleep were calculated using a similar approach. This illustration is a simplification. In reality, we have four sleep cycles and thus more sleep fragments

We separately modelled the survival curves of NREM sleep, REM sleep, and wake. This is illustrated in Fig. 1. NREM fragments were considered terminated if followed by epochs scored as either wake or REM sleep. For the NREM analysis, we excluded NREM fragments with a length below 1 min, to limit the influence of 30-s epoch N1 fragments occurring during wake and REM. REM fragments were terminated if they were followed by epochs either classified as wake or as NREM sleep. Again, we excluded REM fragments with a length below 1 min. Wake fragments were terminated when followed by any epoch scored as sleep, except single N1 epochs (N1 being a subset of NREM sleep). Single N1 epochs during wake were replaced by wake, because they may give a false impression that wake is divided into many shorter fragments. Wake fragments with a length below 1 min were *not* excluded from analysis, because usually the majority of the awakenings during the night is short.

In the survival analysis that was described above, we assumed that single epochs scored as N1 do not interrupt NREM sleep. We also performed an additional analysis, testing such assumption. Here, prior to fitting survival curve, we replaced single N1 epochs occurring during N2 or N3 with single epochs of wake. Subsequently, we proceeded with the analysis as described before.

Theoretically, we could combine all sleep and wake fragments together on the group level using the Kaplan-Meier curves. However, this type of analysis does not take into account the clustering of sleep and wake fragments within participants and is more difficult to use when assessing the combined influence of group and treatment. Therefore, the Kaplan-Meier plots were only used for visual comparison. Instead, we made a parametrization of the survival curves for each individual, using Weibull distributions. Weibull parameters for each of the participants were then compared across group and treatment. A Weibull distribution is characterized by two parameters: a shape parameter (*k*) and a scale parameter (λ). The shape parameter characterizes the shape of the distribution. *k* below 1 indicates that the probability of an event to occur decreases over time. This is often the case for wake fragments, because the majority of the awakenings are very short, and thus the chance to fall asleep again is largest during the first couple of minutes. When the shape parameter is equal to 1, the distribution is exponential. An exponential distribution is the probability distribution when the time between events follows a Poisson process, where events occur at a constant average rate, independently of the time elapsed. An exponential distribution is described only by the scale parameter ʎ, i.e., the event rate. The expected value of an exponentially distributed random variable is 1/ʎ, which in our situation would be equal to the average sleep or wake segment length. To improve the interpretability of the results, we reported the reciprocal of the estimated scale parameters (1/ʎ) in the “Results” section. In a distribution similar to an exponential distribution, a higher reported value of 1/ʎ indicates a longer average segment length and an increased stability.

### Sleep structure over the night

It is possible that sleep and wake dynamics differ over consecutive sleep cycles. This could not be evaluated in our previously described analysis. Therefore, the time course of the proportion of NREM and REM sleep was plotted per treatment condition as a function of time elapsed since sleep onset. The proportion of the sleep stages was defined for each time point as the total number of epochs scored at a certain stage at that designed time point, across all participant from each group, divided by the total number of epochs at that time point. To quantify differences for treatment conditions, additional statistical analysis would be required. However, a detailed analysis of NREM and REM sleep over the night was not within the scope of this article. Therefore, these graphs were used for visual reference only.

### Assessing the amount of sleep misperception

The amount of sleep onset misperception was calculated for each night as the difference between self-reported SOL from the hypnogram and objective SOL, defined as the latency from bedtime until the first epoch scored as sleep from the hypnogram. The amount of TST misperception was calculated as the difference between objective and self-reported subjective TST.

### Estimating subjective sleep onset from sleep fragmentation

**Fig. 2:**
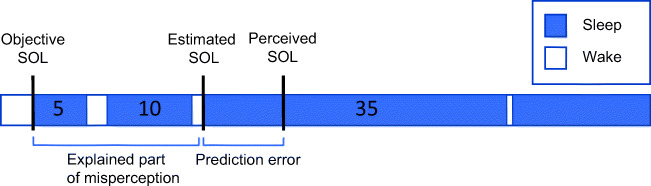
Estimation of the amount of sleep onset misperception according to the sleep length model. Subjective sleep onset was estimated from the hypnogram as the difference between lights off time and the start of the first sleep fragment longer than 30 min. In the figure, this estimate is labelled as “estimated SOL.” Subsequently, the prediction error between the estimated subjective sleep onset and self-reported subjective sleep onset from the sleep diary was calculated. This is referred to as “sleep onset misperception not explained by sleep fragmentation.” Furthermore, the difference between objective sleep onset and estimated subjective sleep onset was calculated. This was referred to as the “explained part of sleep onset misperception”

We used the previously introduced model to determine whether the difference in sleep onset misperception between the zopiclone night and the placebo night could be attributed to predictable changes in sleep fragmentation or to factors not explained by the model (Hermans et al. 2019). In the sleep length model, it was assumed that sleep bouts with a too short length at sleep onset are perceived as wake (Hermans et al. 2019). Thus, the perceived sleep onset was estimated as the start of the first sleep fragment longer than L minutes. Sleep length parameter L was the parameter of the model, i.e., the minimum length a continuous sleep fragment should have in order to be perceived as sleep. Any wake fragment with a duration of at least one 30-s epoch was considered an interruption of sleep. In a previous study, we applied the model to PSG data from people with insomnia and healthy controls, testing different model assumptions (Hermans et al. 2020a). We found a median optimal parameter L of approximately 30–35 min for participants with insomnia, with small variations depending on subgroup characteristics (Hermans et al. 2020a). The optimal parameter L for an individual was referred to as Sleep Fragment Perception Index (SFPI). In the current study, we assigned a reference SFPI of 30 min to all participants and used the model to estimate subjective SOL. The estimated SOL, i.e., the latency until the first uninterrupted sleep fragment longer than 30 min, was subtracted from the objective SOL to obtain an estimate of the amount of sleep onset misperception that can be explained by the model. This procedure is illustrated in Fig. 2. Subsequently, we calculated the prediction error between the estimated subjective sleep onset and actual perceived sleep onset based on the sleep diary. This quantity is equal to the difference between the estimated amount of SOL misperception and actual amount of SOL misperception, because objective SOL is used for both calculations. The prediction error of the model was referred to as “sleep onset misperception not explained by sleep fragmentation” and was compared across groups and treatments. The estimated amount of sleep onset misperception was also compared across groups and treatment conditions.

### Statistical analysis

Statistical analysis was done using R (R Development Core Team 2017). A multi-way ANOVA was used to compare parameters across groups and treatments. We used an alpha value of 0.0167 when comparing survival parameters, to correct for multiple testing (we are comparing survival parameters of REM, NREM, and wake). For the same reason, we used an alpha value of 0.025 when assessing the effect of treatment, group, and group x treatment on sleep misperception of TST and SOL. In the case of a significant effect of group, we used a pairwise post hoc test to find the differences between specific groups.

## Results

Two participants were excluded from the analysis because of (partly) missing PSG data. One of these was from the frequent users group, while the other was from the healthy controls group. Demographic characteristics of participants and use of escape medication by participants from the frequent users group are listed in Table 1.

**Table 1:** Demographic characteristics of participants and use of escape medication

Group	*N*	Age (years)	Sex (%M)	#Participants using escape medication under zopiclone treatment	#Participants using escape medication under placebo treatment
Frequent hypnotics users (*n* = 15)	15	62.1 ± 4.3	8 M; 7F (53)	3	4
Infrequent hypnotics users (*n* = 16)	16	62.3 ± 6.2	8 M; 8F (50)		
Healthy controls (*n* = 15)	15	62.8 ± 4.5	7 M, 8F (47)		

Frequent users who expected difficulties during the three hypnotic-free nights prior to the measurement night were provided escape medication. The escape medication consists of zolpidem at a maximum of one dose of 10 mg per night, to be used only in case of intolerable withdrawal effects

### Sleep survival dynamics

**Fig. 3:**
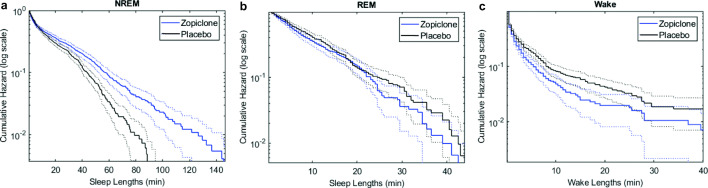
Hazard functions of NREM sleep, REM sleep, and wake for all participants together. All functions are shown on a logarithmic scale. The hazard functions show the probability of the sleep or wake fragment to be terminated during that fragment. (a) Survival curves of NREM sleep. (b) Survival curves of REM sleep. (c) Survival curves of wake

Table 2 reports the Weibull parameters for NREM sleep, REM sleep, and wake. For REM survival curves, no significant effect of either group or treatment was found (Table 2). For NREM sleep, we found a significant effect of treatment for the Weibull scale parameter, which in the case of an exponential distribution would be equal to the average segment length. The NREM scale parameter was significantly smaller during the placebo night than during the zopiclone night. For wake, we found a significant effect of treatment for both Weibull parameters (Table 2). The average segment length of wake was larger during the placebo night compared with the zopiclone night. The shape parameter was larger during the zopiclone night. Figure 3 illustrates the differences of grouped survival curves between the placebo and zopiclone nights, for all participants together. When using the alternative approach to calculate survival curve dynamics of NREM sleep, assuming that N1 sleep also disturbs NREM sleep, no significant effect was found (group x treatment F = 1.37, *p* = 0.26; treatment F = 2.69, *p* = 0.10; group F = 1.18, *p* = 0.31).

**Table 2:** Parameters of the Weibull distribution for the sleep and wake survival analysis per group and treatment condition (mean ± standard deviation)

		*Participant groups*			*Multi-way ANOVA*		
		*Frequent users*	*Infrequent users*	*Healthy controls*	*Treatment*	*Group*	*Treatment x group*
*REM Weibull scale, 1/ʎ*	*Placebo*	*13.5 ± 5.3*	*15.4 ± 8.8*	*12.7 ± 4,5*	*F = 0.99,* *p = 0.32*	*F = 0.83,* *p = 0.44*	*F = 0.52,* *p = 0.60*
	*Zopiclone*	*11.0 ± 5.3*	*13.3 ± 7.1*	*13.3 ± 5.2*			
*REM Weibull shape*	*Placebo*	*1.5 ± 0.57*	*2.4 ± 1.3*	*1.5 ± 0.40*	*F = 0.34,* *p = 0.56*	*F = 2.98,* *p = 0.06*	*F = 0.15,* *p = 0.86*
	*Zopiclone*	*1.8 ± 1.43*	*2.3 ± 2.1*	*1.8 ± 1.1*			
*NREM Weibull scale 1/ʎ*	*Placebo*	*17.3 ± 8.1*	*15.3 ± 3.8*	*17.8 ± 5.0*	*F = 8.89,* ***p < 0.01***	*F = 1.16,* *p = 0.32*	*F = 0.44,* *p = 0.65*
	*Zopiclone*	*20.0 ± 10.8*	*20.8 ± 9.2*	*24.1 ± 8.2*			
*NREM Weibull shape*	*Placebo*	*0.98 ± 0.19*	*0.93 ± 0.09*	*0.89 ± 0.13*	*F = 3.32,* *p = 0.07*	*F = 0.21,* *p = 0.81*	*F = 1.11,* *p = 0.34*
	*Zopiclone*	*0.85 ± 0.17*	*0.86 ± 0.25*	*0.89 ± 0.19*			
*Wake Weibull scale 1/ʎ*	*Placebo*	*3.6 ± 1.8*	*2.8 ± 1.6*	*3.3 ± 3.1*	*F = 11.49,* ***p < 0.01***	*F = 1.48,* *p = 0.23*	*F = 0.36,* *p = 0.70*
	*Zopiclone*	*2.4 ± 1.0*	*1.9 ± 0.8*	*1.5 ± 0.8*			
*Wake Weibull shape*	*Placebo*	*0.87 ± 0.23*	*0.94 ± 0.29*	*0.93 ± 0.43*	*F = 7.22,* ***p < 0.01***	*F = 3.08,* *p = 0.05*	*F = 2.46,* *p = 0.09*
	*Zopiclone*	*0.95 ± 0.25*	*1.20 ± 0.62*	*1.77 ± 1.47*			

Parameters were reported as the inverse of the scale parameter (1/ʎ) to improve the interpretability of the results. In case of a close to exponential distribution (k close to 1), the inverse of the scale parameter 1/ʎ is equivalent to the mean duration of a sleep or wake fragment. Therefore, a higher value of 1/ʎ indicates a larger stability of that sleep or wake stage

### Proportions of sleep stages over the night

**Fig. 4:**
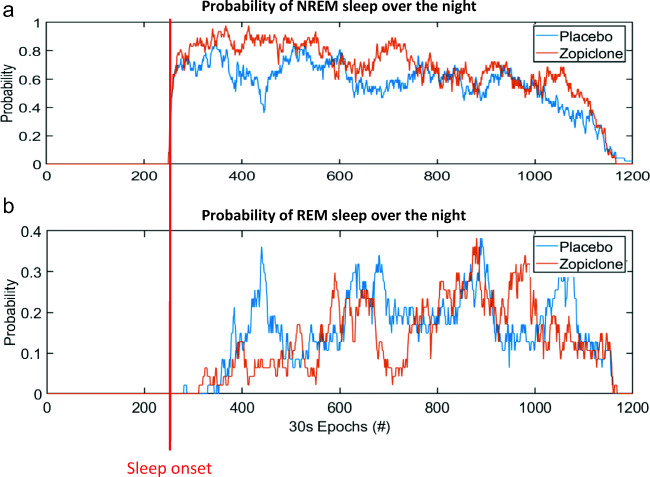
Proportions of sleep stages over the course of the night across all participants. The first epoch of sleep has been aligned between participants, to make it start at the same point in time. (a) Combined proportions of N2 and N3 sleep. (b) Proportions of REM sleep

Figure 4 shows the proportions of NREM sleep and REM sleep over the night for each of the two treatment conditions. Visually, a clear difference of the distribution of REM sleep over the night can be observed between treatment conditions. During the placebo night, a clear peak of REM sleep is visible after approximately 1 h. During the zopiclone night, the first peak of REM sleep seems largely absent.

### Sleep misperception

**Fig. 5:**
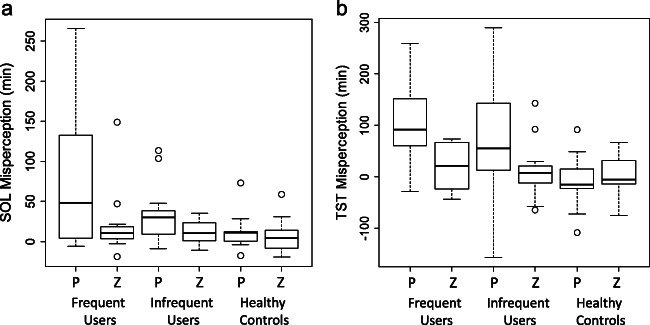
The actual amount of sleep state misperception during the placebo and the zopiclone night, summarized per group and treatment condition. *P* = placebo and *Z* = zopiclone. (a) Sleep onset misperception, i.e., the difference between self-reported subjective SOL (sleep diary) and objectively measured SOL. (b) Misperception of TST, calculated as the difference between self-reported subjective TST and objectively measured TST

Figure 5a shows the actual amount of sleep onset misperception, i.e., the difference between self-reported subjective SOL and objectively measured SOL, during the placebo and the zopiclone night, summarized per group and treatment condition. We found significant effects for both group and treatment, but no interaction effect (Table 3). Pairwise post hoc testing indicated a significant difference between frequent users and healthy controls (Mann-Whitney U = 652, *p* = 0.02) and between infrequent users and healthy controls (Mann-Whitney U = 587.5, *p* = 0.02). For misperception of TST (Fig. 5b), again a significant effect was found for group and treatment (Table 3). Pairwise testing indicated a significant difference between frequent users and healthy controls (Mann-Whitney U = 655, *p* < 0.001) and between infrequent users and healthy controls (Mann-Whitney U = 638, *p* = 0.03).

**Table 3:** Parameters of SOL and TST misperception per group and treatment condition (average ± standard deviation)

		*Participant groups*			*Multi-way ANOVA*		
		*Frequent users*	*Infrequent users*	*Healthy controls*	*Treatment*	*Group*	*Treatment x group*
*Actual amount of SOL misperception (minutes)*^*a*^	*Placebo*	*75.5 ± 82.9*	*33.4 ± 33.2*	*15.2 ± 25.6*	*F = 10.80,* ***p < 0.01***	*F = 6.08,* ***p < 0.01***	*F = 2.49,* *p = 0.09*
	*Zopiclone*	*19.5 ± 38.6*	*12.3 ± 13.3*	*5.5 ± 20.6*			
*Actual amount of TST misperception (minutes)*^*b*^	*Placebo*	*19.4 ± 42.6*	*11.2 ± 50.1*	*− 7.1 ± 47.3*	*F = 10.41,* ***p < 0.01***	*F = 6.87,* ***p < 0.01***	*F = 4.05,* *p = 0.02*
	*Zopiclone*	*104.4 ± 82.2*	*76.7 ± 113.5*	*3.7 ± 39.8*			
*Estimated amount of SOL misperception (minutes)*^*c*^	*Placebo*	*18.1 ± 17.6*	*9.1 ± 12.9*	*9.4 ± 13.5*	*F = 16.1,* ***p < 0.001***	*F = 1.70,* *p = 0.19*	*F = 0.60,* *p = 0.55*
	*Zopiclone*	*57.6 ± 72.9*	*47.5 ± 47.8*	*29.2 ± 30.5*			
*Prediction error of misperception (minutes)*^*d*^	*Placebo*	*18.6 ± 73.1*	*− 13.4 ± 61.3*	*− 11.3 ± 20.7*	*F = 0.20,* *p = 0.65*	*F = 1.62,* *p = 0.20*	*F = 1.01,* *p = 0.37*
	*Zopiclone*	*4.7 ± 34.2*	*3.9 ± 20.2*	*− 3.1 ± 24.8*			

^a^The difference between self-reported subjective SOL obtained from the sleep diary and objective SOL

^b^The difference between subjective TST obtained from the sleep diary and objective TST

^c^The difference between subjective SOL estimated from the hypnogram and objective SOL

^d^The difference between estimated amount of SOL misperception and actual amount of SOL misperception

### Estimated SOL misperception and prediction error

**Fig. 6:**
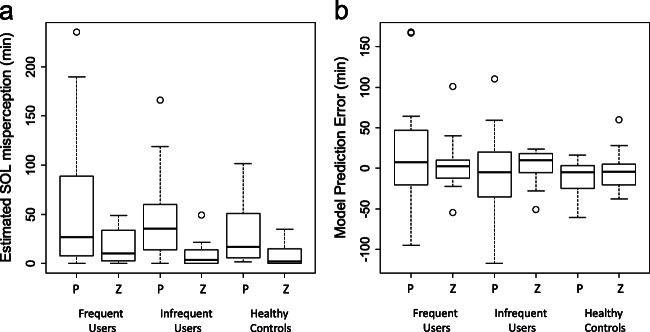
Results of using the model to estimate the subjective SOL for individual patients, shown per group and treatment condition. *P* = placebo and *Z* = zopiclone. (a) Estimated amount of SOL misperception, defined as the difference between objective and estimated subjective SOL. Subjective SOL was estimated as the latency until the start of the first uninterrupted sleep fragment longer than 30 min. (b) Prediction error, calculated as the difference between estimated sleep onset misperception and the actual self-reported sleep onset misperception

Figure 6 shows the results of using the sleep length model to estimate the amount of SOL misperception, summarized per group and treatment condition. There was a significant effect of treatment in the estimated amount of SOL misperception (Table 3; Fig. 6A). No significant group effect was found. The prediction error of SOL misperception did not show any significant difference across group or treatment (Table 3; Fig. 6B). However, when comparing the variance of all placebo nights to the variance of all zopiclone nights, we found that variances were significantly larger during the zopiclone nights (Levene’s test, center = median, F = 7.09, *p* < 0.01).

## Discussion

We analyzed experimental manipulations of sleep in a pharmacological intervention protocol. The aim of the study was to explore potential mechanisms of sleep fragmentation that influence alterations of perceived sleep quality and quantity observed using a pharmacological manipulation. Results indicate an increased stability of NREM sleep and a decreased stability of wake during the zopiclone night compared with the placebo night. Additionally, we used a previously proposed model to estimate subjective sleep onset from the hypnogram based on sleep fragmentation. Both the so-obtained estimated amount of SOL misperception and the actual amount of SOL misperception were significantly lower during the zopiclone night.

When fitting a Weibull distribution to the NREM sleep segments, we found that the inverse of the scale parameter 1/ʎ was significantly higher during the zopiclone night, while the shape parameter was unaltered. The altered scale parameter suggests that fragments of NREM sleep overall have a higher probability to “survive” during the zopiclone night compared with the placebo night. Thus, NREM sleep seems more stable during the zopiclone night. This finding might be partly explained by the increased percentage of slow wave sleep that was reported during the zopiclone night (Leufkens et al. 2014), because from previous experimental research, it appears that the continuity of deep sleep is better protected compared with lighter sleep (Rechtschaffen et al. 1966). The unaltered Weibull shape parameter suggests that the distribution of the length of the NREM sleep fragments was not altered. Interestingly, when repeating the analysis with the alternative assumption that NREM sleep is not only disturbed by wake and REM epochs, but also by epochs scored as N1, we did not find a significant difference between treatment conditions. According to R&K guidelines, epochs of N1 are often scored when arousals during N2 sleep are observed (Rechtschaffen and Kales 1968). Therefore, our present results may indicate that the distribution and number of arousals were unaltered across treatment conditions. We may speculate that sleep disturbances by awakenings were more important for the quality of the sleep than arousals. This hypothesis is supported by the fact that the model of subjective sleep onset adequately predicted a change of sleep onset misperception, without taking into account N1 sleep or arousals.

Additionally to alterations in dynamics of NREM sleep, we also found differences in both the wake scale and shape parameters between treatment conditions. The altered scale parameter during the zopiclone night indicates a decreased stability of wake fragments, i.e., participants fell asleep sooner after awakening. This finding explains the shorter WASO and longer TST during the zopiclone night, as previously reported (Leufkens et al. 2014). The larger scale parameter during the zopiclone night probably resulted from a decreased percentage of long awakenings. The fact that not only scale parameters but also shape parameters can differ across treatment conditions provides support for our approach to use a Weibull distribution instead of an exponential distribution. For the current dataset, lower percentages of REM sleep were reported during the zopiclone night (Leufkens et al. 2014). Indeed, the effects on REM sleep density are commonly reported for zopiclone (Hemmeter et al. 2000; Kanno et al. 1993; Kim et al. 1993). Survival curve analysis did not indicate a decreased stability of REM sleep, leading to the conclusion that only the probability of entering REM sleep was reduced. Indeed, plotting the probability of REM sleep over the night suggests an absence of the first peak of REM sleep during the zopiclone night. Since a detailed analysis of NREM and REM sleep over the night was not within the scope of this article, we did not perform statistical analysis on this.

Since using hypnotics can result in large changes of many aspects of sleep architecture, it is difficult to precisely indicate which of these changes are associated with sleep quality. Therefore, we used a modelling approach to demonstrate the relationship between sleep fragmentation and sleep onset misperception, which may be an expression of impaired objective sleep quality at the beginning of the night. We found a lower amount of predicted sleep onset misperception during the zopiclone night, as well as a lower amount of actual sleep onset misperception. In contrast, the prediction error of the model did not differ between treatment conditions. These results suggest that a considerable part of the difference of sleep onset misperception between treatment conditions can be explained by predictable alterations of sleep fragmentation at the beginning of the night. Importantly, the model to estimate subjective sleep onset was developed from another night of measurement, partly with the same study participants (Hermans et al. 2019). This may explain why the parameter of 30 min seemed to fit so well in the current study. However, the model was validated in an independent and larger study sample with younger participants from a different sleep laboratory. Also in that case, the parameter of 30 min was proven to be applicable outside the initial study population (Hermans et al. 2020a). Although the prediction error of the model, i.e., the unexplained component of sleep onset misperception, did not differ significantly between treatment conditions, its variation was larger during the placebo night. Therefore, it is possible that zopiclone also influences components of sleep onset misperception not explained by the model, but to a lesser extent. Other mechanisms of influence could include anterograde amnesia, which is a side effect reported for zopiclone (Sanofi-aventis Inc 2018; World Health Organization 2006).

The current finding that decreased stability of NREM sleep may be associated with impaired sleep quality is consistent with previous modelling results, which indicate that the length of sleep fragments at the beginning of the night is important for the perception of the sleep onset (Hermans et al. 2020a). Furthermore, in the current study, a decreased stability of wake was indicated as a possible parameter reflecting impaired sleep quality. However, in previous research, we found that the length of wake fragments interrupting sleep was not of great importance for the perception of the sleep onset, mainly because the majority of the awakenings were very short (Hermans et al. 2020a). Additionally, in earlier research, the subtype of insomnia with short objective sleep duration was not associated with sleep state misperception (Fernandez-Mendoza et al. 2011). Considering that long awakenings often co-occur with a short sleep duration, it is possible that the current findings of altered wake survival parameters are not connected with sleep (onset) misperception. However, it is possible that the length of the wake fragments is associated with other components of sleep quality. Next to altered parameters of NREM sleep and wake, a third finding of sleep architectural changes during the zopiclone night was a delayed latency until REM sleep. REM sleep latency is not incorporated in our model of sleep onset misperception and therefore is not part of the explained part of sleep onset misperception. However, we cannot exclude the possibility that the delay of REM sleep during the zopiclone night influenced sleep onset misperception as part of the altered variance of the unexplained part of the sleep onset model.

A limitation of this research is that we used the 30-min parameter to estimate subjective sleep onset in all participants, because estimating optimal parameters for individuals would require multiple nights of data per subject and per treatment condition. Previous research yielded an optimal parameter of approximately 20 min for healthy controls (Hermans et al. 2020a). Thus, by estimating subjective sleep onset as the first sleep fragment longer than 30 min, we probably exaggerated the influence of sleep fragmentation in this group. However, the number of sleep fragments with a length between 20 and 30 min at sleep onset was very limited, and thus, the exact choice of the parameter would probably not heavily influence the results. As another limitation, the current survival analysis was based on R&K scoring rules. Therefore, results may slightly differ from PSGs scored according to the AASM guidelines. However, based on research by Moser at al. comparing the AASM and R&K guidelines, we expect that the percentage of epochs scored as N1 during NREM sleep may be influenced by the scoring rules used, while differences for wake epochs after sleep onset may be very limited (Moser et al. 2007). Therefore, we expect that the scoring guidelines used will only influence the results of the approach in which we assumed that N1 stages interrupt NREM sleep, while the analysis in which we only considered awakenings seems most promising for further research. A third limitation was that patients had a fixed bedtime in this study. It is possible that misalignments between the participant’s usual bedtime and the fixed bedtime influenced the results for sleep onset misperception. However, since both measurement nights had the same fixed bedtime, we do not expect that the conclusions on the effect of treatment will be affected. Still, it is possible that a confounding effect was present for the group comparisons. This highlights the additional need to study survival parameters in larger study samples.

In this study, improvements of subjective sleep quality and quantity co-occurred with decreased stability of NREM sleep and increased stability of wake over the entire night, while no alterations of REM stability were observed. In a similar protocol with multiple nights of zolpidem and multiple nights of placebo, similar improvements of perceived sleep quantity and quality were found during the zolpidem nights (Kryger et al. 1991). During the zolpidem nights, the latency to persistent sleep became shorter (Kryger et al. 1991), pointing towards the possibility that our model would also have estimated a lower amount of sleep onset misperception in this case. However, whole-night differences of number of awakenings and WASO were not found (Kryger et al. 1991). This finding could possibly be related to the shorter half-life time of zolpidem compared with zopiclone, as well as to the possibility that survival parameters are better suitable to express relevant aspects of sleep fragmentation.

From the present data, we cannot conclude whether the altered sleep dynamics we found are specific for the influence of zopiclone in elderly people, or if they also play a role in younger people not using hypnotics. Larger datasets are required to examine if differences of NREM and wake dynamics can be found between people with insomnia and healthy participants. Such differences were not found in our study. However, based on the results of a simplified sample size calculation, comparing two independent means (Dhand and Khatkar 2014) and assuming means and standard deviations similar to the ones found in this study, we conclude that a difference between a NREM scale parameter of 20 and 15 min with a standard deviation of 8 min would require a sample size of 41 participants with insomnia and 41 healthy controls. If the survival curve of NREM is close to mono-exponential, this would correspond to a difference between three and four awakenings per hour, which in our opinion could already be a clinically significant difference. Therefore, we conclude that, at least for the NREM scale parameter, our sample size was probably not large enough to detect clinically meaningful differences between groups. Previous research does indicate that differences of sleep dynamics may exist between people with insomnia and healthy controls (Roth et al. 2016). Furthermore, it has been shown that patients with insomnia have a higher probability of transitioning from stage N2 to N1 or wakefulness compared with healthy controls and a decreased stability of stage N2 (Wei et al. 2017). In light of the current results, we can speculate that these differences may indeed represent differences of sleep quality between patients with insomnia and controls.

In this study, we treated impaired subjective sleep quality, sleep onset misperception, and misperception of TST as different expressions of the same objectively measurable sleep quality. However, there might be different aspects of sleep quality that are influenced by different parameters. For example, currently we do not know if sleep onset misperception and misperception of TST share the same mechanisms. It is difficult to separately assess misperception of SOL and TST, because TST misperception is influenced by SOL misperception. Therefore, we would like to stress the importance of specifically asking study participants about their subjective WASO. When studying larger groups of patients with insomnia, it would be interesting to use interindividual differences between components of sleep quality to disentangle different mechanisms. For example, it is possible that part of patients with insomnia predominantly experiences sleep onset misperception, while other patients mainly have complaints of misperception of WASO, or impaired subjective sleep quality without marked sleep state misperception. Within the patient population, these different subtypes could be compared regarding survival parameters. As such, the survival parameters identified in this study could be possibly used as a tool for understanding mechanisms of impaired sleep quality in specific subtypes of sleep problems. Additionally, dividing sleep onset misperception into a component explained by sleep fragmentation and an unexplained component can also present valuable opportunities for research into treatment interventions.
